# SIRT6 Activator UBCS039 Inhibits Thioacetamide-Induced Hepatic Injury In Vitro and In Vivo

**DOI:** 10.3389/fphar.2022.837544

**Published:** 2022-04-20

**Authors:** Fangzhou Jiao, Zongwei Zhang, Hongtu Hu, Yongxi Zhang, Yong Xiong

**Affiliations:** ^1^ Department of Infectious Diseases, Zhongnan Hospital of Wuhan University, Wuhan, China; ^2^ Department of Nephrology, Renmin Hospital of Wuhan University, Wuhan, China

**Keywords:** SIRT6, thioacetamide, acute liver failure, inflammation, oxidative stress

## Abstract

SIRT6 has been reported to have multiple functions in inflammation and metabolism. In the present study, we explored the regulatory effects and mechanisms of SIRT6 in thioacetamide (TAA)-induced mice acute liver failure (ALF) models. The SIRT6 activator UBCS039 was used in this animal and cell experiments. We observed that UBCS039 ameliorated liver damage, including inflammatory responses and oxidative stress. Further study of mechanisms showed that the upregulation of SIRT6 inhibited the inflammation reaction by suppressing the nuclear factor-κB (NF-κB) pathway in the TAA-induced ALF mice model and lipopolysaccharide-stimulated macrophages. In addition, the upregulation of SIRT6 alleviated oxidative stress damage in hepatocytes by regulating the Nrf2/HO-1 pathway. These findings demonstrate that pharmacologic activator of SIRT6 could be a promising target for ALF.

## Introduction

Acute liver failure (ALF) is an unusual clinical syndrome characterized by massive hepatocyte necrosis without pre-existing liver diseases (Jambekar et al., 2011). ALF is a high-mortality liver disease with multiple devastating complications. The etiologies of ALF are complicated and diverse, including viral hepatitis, toxins, hepatic ischemia reperfusion injury, and metabolic disorders (Lin et al., 2018). To date, there is no effective drug therapy for ALF, except liver transplantation (Lee 2012). However, liver transplantation is limited by rapidly progressive liver disease, high surgery costs, and shortage of donor livers. It is particularly urgent to develop novel therapeutic strategies for ALF. A critical feature of ALF is the inflammatory response of liver tissues such as the massive infiltration of inflammatory cells and cytokine storm. Hence, inhibition and regulation of liver inflammation might be a promising strategy for the prevention and treatment of ALF.

Hepatic macrophages are important components of the host defense system against pathogens and liver toxins in the liver (Possamai et al., 2014). In several animal ALF models, local hepatic injury is characterized by the increased number of macrophages (Holt et al., 2008). Nuclear factor-κB (NF-κB) is a critical transcription factor that mediates the inflammatory process by regulating the transcription of multiple inflammation-related cytokines (Sun 2017). Apart from hepatic inflammation, oxidative stress has been confirmed as another critical pathogenic factor involved in hepatic cell injury. A previous study has reported that an antioxidant (N-acetylcysteine amide) alleviated liver injury by inhibiting oxidative stress in ALF mice (Khayyat et al., 2016). In summary, both inflammation and oxidative stress are associated with the progress of ALF.

Sirtuins are a special class of the histone deacetylase family, which can regulate the acetylation of nicotinamide adenine dinucleotide (NAD^+^). Sirtuins are also considered as critical enzymes involved in various biological functions, especially in metabolism and inflammation (Vachharajani et al., 2016). SIRT6, a nuclear protein and a special member of the sirtuin family, has the activity of ADP-ribosyl transferase and NAD^+^-dependent deacetylase and is involved in multiple biological functions including life span, inflammation, metabolism, and cancer. Increasing studies have confirmed the beneficial role of SIRT6 in inflammatory diseases (Liu G. et al., 2021). Therefore, SIRT6 might have a role in regulatory mechanisms of the inflammatory process, which participate in the progress of ALF.

A previous *in vivo* study has reported that SIRT6 siRNA increased the expression of pro-inflammatory cytokines in human umbilical vein endothelial cells (HUVECs) with LPS treatment (Lappas 2012). Another study showed that SIRT6 activator UBCS039 reduced the expressions of iNOS and NF-κB in LPS/IFN-γ-stimulated macrophages (Zou et al., 2021). Similarly, a recent study found that UBCS039 decreased the expressions of TNF-a, IL-1b, and IL-6 in LPS-challenged bone marrow-derived macrophages (BMDMs) (Wang et al., 2022). These research works supported the anti-inflammatory characteristics of SIRT6. Recently, one study has shown that hepatic SIRT6 was downregulated in acetaminophen (APAP)-induced ALF, and SIRT6 knockdown exacerbated hepatocyte death and oxidative stress in AML12 cells after APAP stimulation (Zhou et al., 2021). I*n vitro* studies indicated the protective effect of SIRT6 on liver injury. However, there has been no related experimental study of SIRT6 in the animal ALF model so far. In the present study, the SIRT6 selective activator UBCS039 was used to explore the protective effect and the potential mechanism in thioacetamide (TAA)-induced hepatic injury *in vitro* and *in vivo.*

## Materials and Methods

### Reagents

UBCS039, tert-butylhydroquinone (TBHQ), and ammonium pyrrolidinedithiocarbamate (PDTC) were obtained from MedChemExpress (China). Thioacetamide (TAA) was obtained from Sigma (United States). SIRT6 was purchased from ProteinTech (China). Phospho-NF-κB-p65 (#3033), NF-κB-p65 (#8242), phospho-IκBα (#2859), IκBα (#4812), TNF-α (#11948), IL-1β (#12426), Bax (#5023), Bcl-2 (#15071), cleaved caspase 3 (#9664), Nrf2 (#12721), HO-1 (#43966), and GAPDH (# 5174) were obtained from Cell Signaling Technology (United States). The list of chemical and reagents is shown in Table 1.

**TABLE 1:** List of chemicals and reagents.

Reagent	Application	Catalog	Manufacturer
UBCS039	SIRT6 activator	358721-70-7	MedChemExpress (China)
tert-Butylhydroquinone (TBHQ)	Nrf2 activator	1948-33-0	MedChemExpress (China)
Ammonium pyrrolidinedithiocarbamate (PDTC)	NF-κB inhibitor	5108-96-3	MedChemExpress (China)
Thioacetamide (TAA)	-	163678	Sigma-Aldrich (United States)
Lipopolysaccharide (LPS)	-	L6529	Sigma-Aldrich (United States)
SIRT6	Antibody	13572-1-AP	ProteinTech (China)
Phospho-NF-κB-p65	Antibody	#3033	Cell Signaling Technology (United States)
NF-κB-p65	Antibody	#8242	Cell Signaling Technology (United States)
Phospho-IκBα	Antibody	#2859	Cell Signaling Technology (United States)
IκBα	Antibody	#4812	Cell Signaling Technology (United States)
TNF-α	Antibody	#11948	Cell Signaling Technology (United States)
IL-1β	Antibody	#12426	Cell Signaling Technology (United States)
Bax	Antibody	#5023	Cell Signaling Technology (United States)
Bcl-2	Antibody	#15071	Cell Signaling Technology (United States)
Cleaved caspase 3	Antibody	#9664	Cell Signaling Technology (United States)
Nrf2	Antibody	#12721	Cell Signaling Technology (United States)
HO-1	Antibody	#43966	Cell Signaling Technology (United States)
GAPDH	Antibody	# 5174	Cell Signaling Technology (United States)

### Animal Experiments

Six-week-old male C57BL/6 mice (weighing 20–25 g) were obtained from Beijing Vital River Laboratory Animal Technology (Beijing, China). All animal protocols were approved by the Animal Care and Use Committee of Wuhan University. Thirty experimental mice were randomly divided into the following three groups (*n* = 10 each group): control group, model group, and UBCS039 group. As described in our previous study, the ALF model was established by the intraperitoneal injection of 300 mg/kg TAA (Jiao et al., 2019). In the UBCS039 group, mice were given UBCS039 (50 mg/kg) by intraperitoneal injection 2 h before TAA administration. The dosage of UBCS039 was selected according to previous dosage experiments. In the control group, mice were administered with the same doses of normal saline. At the end of the experiment (24 h after TAA injection), all mice were killed after anesthesia. Then, the liver and blood sample were harvested.

### Blood Measurements

An automatic biochemistry analyzer (Hitachi, Japan) was used to measure the levels of alanine aminotransferase (ALT) and aspartate aminotransferase (AST) in each blood sample. The serum contents of TNF-α, IL-6, and IL-1β were detected by using the ELISA kit (Jianglai Biotech, Shanghai).

### Assessment of Malondialdehyde and Glutathione

Malondialdehyde (MDA) and glutathione (GSH) were used to evaluate the degree of liver oxidative stress. The MDA and GSH kits were obtained from Jianglai Biotech (Shanghai). The changes in the MDA and GSH levels were measured according to the manufacturer’s instructions book (Jianglai Biotech, Shanghai).

### Liver Histological Analysis

The whole liver was taken and photographed after killing the mice. A part of the liver tissues were fixed in 10% formaldehyde, embedded into paraffin after dehydration, and sliced into 5-µm-thick sections. The section was stained with hematoxylin and eosin (H&E). The pathological morphology of liver tissue was observed under the light microscope (Olympus, Japan). As described in the previous study, the liver histological score was used to estimate liver injury (Jiao et al., 2021).

### Immunofluorescence Staining

Liver tissue sections were then fixed with 10% bovine serum albumin (BSA) for 1 h. After washing three times with PBS, the sections were stained with an anti-F4/80 antibody (1:100 dilutions; Abcam) and an anti-SIRT6 antibody (1:100 dilutions; ProteinTech) for 24 h. Then, the sections were incubated with a biotinylated secondary antibody. The images were viewed and photographed under a fluorescence microscope.

### TUNEL Staining

TUNEL staining was used to measure the cell death in liver tissues. According to the manufacturer’s instructions (Roche, United States), the sections were incubated with proteinase K at room temperature for 15 min. After washing three times, the sections were incubated with TUNEL solution. The staining results were viewed and photographed under a fluorescence microscope.

### Cell Culture and Intervention

The liver cell lines LO2 and macrophage RAW264.7 were purchased from China Center for Type Culture Collection (CCTCC). The 2 cell lines were cultured in DMEM containing 10% fetal bovine serum and 1% penicillin/streptomycin. The cell incubator was maintained at 5% CO_2_ at 37 C. LPS (L2880, Sigma) was used to establish the *in vitro* inflammation model in RAW264.7. The LO2 cells were treated with TAA solution to induce liver cell injury *in vitro* (Lim et al., 2013).

### CCK-8 Experiment

Cell viability of the LO2 cells or RAW264.7 cells was determined by Cell Counting Kit-8 assay, according to the manufacturer’s instructions (# CK04, Dojindo, Japan). Briefly, the cells were cultured in 96-well plates at a density of 1×10^4^ cells/well, and then the cells were exposed to different concentrations of UBCS039 or TAA for 24 h. After treatment, the culture medium was replaced with 10 μl CCK-8 dye and 90 μl medium for 1 h at 37°C. The amount of formazan dye generated by cellular dehydrogenase activity in cells was measured at a wavelength of 450 nm.

### SiRNA Transfection

SiRNA was purchased from RiboBio (Guangzhou, China); the human SIRT6 siRNA sequence is 5’-GGA​AGA​ATG​TGC​CAA​GTG​T-3’, and the mouse SIRT6 siRNA sequence is 5’-GCC​GTC​TGG​TCA​TTG​TCA​A-3’. LO2 cells or RAW264.7 cells (1 × 10^5^ cells/well) were seeded in a six-well plate, and siRNA transfection was performed until the cells reached 70% confluency. The cells were transfected with scramble siRNA or SIRT6 siRNA in the presence of transfection reagent Lipofectamine 2000. After 24 h, all cells were harvested for protein and mRNA extractions. The protein levels and gene levels of SIRT6 were measured to evaluate SIRT6 knockdown efficiency.

### Assessment of ROS

Intracellular ROS of LO2 cells was detected by using the ROS Assay Kit, according to the manufacturer’s instructions (S0033S, Beyotime, China). Briefly, after cell intervention, the cell medium was replaced with a new medium containing 10 μM DCFH-DA. After 30-min incubation at 37°C, the cells were washed three times with a serum-free medium for removing extra DCFH-DA. Then, the cells were imaged under a fluorescence microscope.

### Western Blotting

Liver tissues and cells were lysed with protein extraction buffer containing RIPA, PMSF, and phosphatase inhibitors. Protein samples were separated by using 10% SDS-PAGE, transferred to a polyvinylidene fluoride membrane, fixed with 5% BSA, and incubated with primary antibodies overnight at 4°C. After incubation with primary antibodies, including anti-SIRT6, phospho-NF-κB-p65, NF-κB-p65, phospho-IκBα, IκBα, TNF-α, IL-1β, Bax, Bcl-2, cleaved caspase 3, Nrf2, HO-1, and GAPDH, the membranes were washed three times with TBS-T buffer, followed by the HRP-conjugated secondary antibody for 1 h at room temperature. After washing, the membranes were visualized by using ECL reagents. The values of bands were analyzed by using the ChemiDocTM MP Imaging System (Bio-Rad, United States).

### Quantitative RT-PCR Assay

The total RNA of cells was extracted by using TRIzol reagent. The concentration of RNA was measured by a NanoDrop spectrophotometer. Then, RNA was converted to cDNA by using the PrimeScript RT-PCR kit. The cDNAs were amplified by using the SYBR Green Kit on a 7500 Real-Time PCR System. The PCR amplification protocol and calculation method of gene expression were performed as per our previous study (Jiao et al., 2021). The primer sequences were as follows: SIRT6, forward, 5‘- CAG TAC GTC AGA GAC ACG GTTG -3’; reverse, 5‘- GTC CAG AAT GGT GTC TCT CAGC-3’; GAPDH, forward, 5‘- CAT CAC TGC CAC CCA GAA GAC TG -3’; reverse, 5‘- ATG CCA GTG AGC TTC CCG TTC AG -3’.

### Statistical Analysis

Data were represented as mean ± standard deviation. The statistical differences of two groups were calculated by using Student’s *t*-test. The statistical data among multiple groups were analyzed by one-way ANOVA. The statistical process was performed by SPSS 12.0 software. The statistical differences were considered if the *p* value <0.05.

## Results

### The Protein Expression of SIRT6 in Liver Tissues Was Reduced in Acute Liver Failure Mice

**FIGURE 1:**
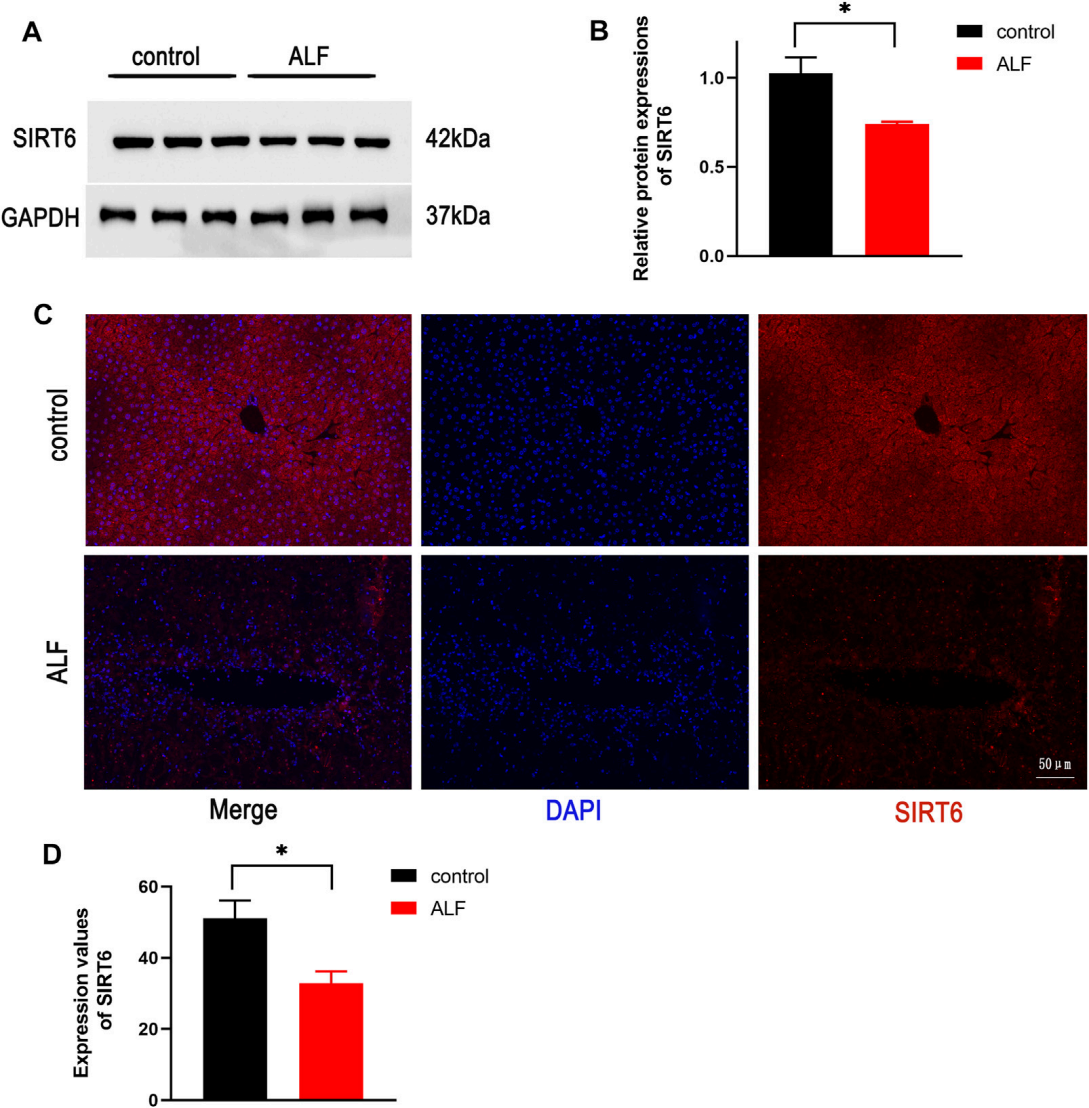
SIRT6 expression of liver tissue in the TAA-induced ALF model in mice. **(A)** Western blot analysis of SIRT6 levels in TAA-induced ALF mice. **(B)** Densitometric analysis of SIRT6. **(C)** Immunofluorescence staining of SIRT6 in liver tissue. **(D)** Quantitative value of fluorescence. *p < 0.05, compared with the control group. *n* = 5 per group. Data are represented as mean ± SD.

We first explored the SIRT6 levels in the liver after TAA administration. The protein expression in liver tissues examined by Western blotting revealed that SIRT6 was downregulated in ALF mice (Figures 1A,B). Immunofluorescent staining showed a similar conclusion that the expressions of SIRT6 were significantly decreased in ALF mice in comparison with normal mice (Figures 1C,D). These results indicated that the decrease in SIRT6 was related to liver injury.

### UBCS039 Attenuated Liver Damage in Acute Liver Failure Mice

**FIGURE 2:**
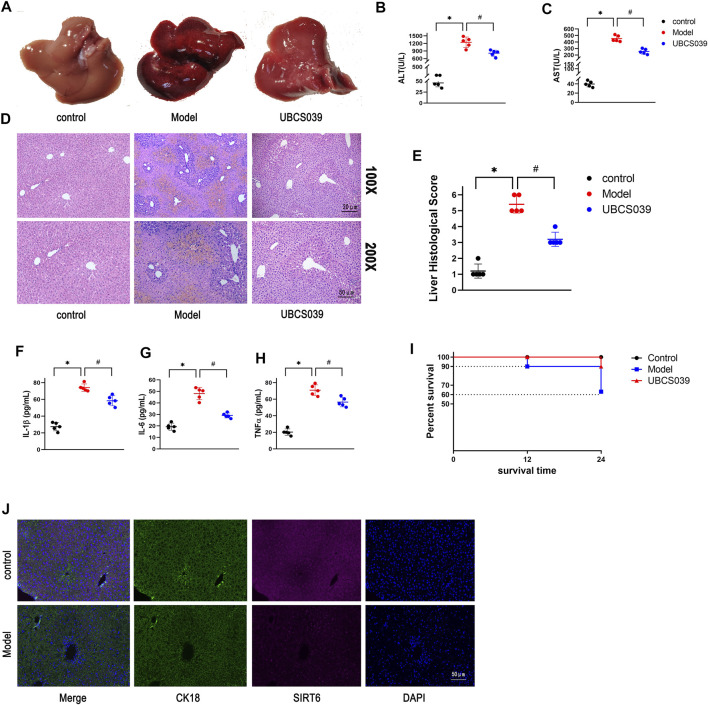
Effects of UBCS039 on liver damage in the ALF model in mice. **(A)** Change of liver morphology. **(B)** ALT levels. **(C)** AST levels. **(D)** HE-stained liver tissues. **(E)** Histological score of liver tissue. ELISA measured the serum IL-1β **(F)**, IL-6 **(G)**, and TNF-α **(H)** in each group. **(I)** Survival curves of mice in each group. **(J)** Immunofluorescent staining of liver sections with anti-CK18 (green) and anti-SIRT6 (red). *p < 0.05, compared with the control group. #*p* < 0.05, compared with the model group. *n* = 5 per group. Data are represented as mean ± SD.

Liver morphology showed the serious liver injury in ALF mice, and there was no significant change in the control and UBCS039 groups (Figure 2A). The serum ALT and AST levels were measured to evaluate the degree of liver function damage. The levels of ALT and AST were both obviously elevated in model mice, whereas UBCS039 administration decreased the serum ALT and AST levels in ALF (Figures 2B,C). HE staining revealed that the model group has more serious structural destruction of hepatic tissue with a large area of necrosis and increased numbers of infiltrative inflammatory cells compared to the control group (Figure 2D). However, the area of hepatic necrosis and injury was decreased in the UBCS039 group (Figure 2D). Consistently, UBCS039 reduced the liver score of histology compared with model groups (Figure 2E). ELISA showed an increase in inflammatory cytokines including TNF-α, IL-6, and IL-1β in model mice compared with normal mice. Similarly, UBCS039 reduced these cytokine levels (Figures 2F–H). In addition, survival rate analyses revealed that the model group at 24 h had 60% and UBCS039 group had 90% survival rate (Figure 2I). Finally, in order to measure the expression of SIRT6 on hepatocytes, the immunofluorescent staining of liver sections with anti-CK18 (hepatocyte surface markers) and anti-SIRT6 was performed (Figure 2J). The staining result showed the decrease in SIRT6 in hepatocytes in ALF liver tissues. The aforementioned data indicated that UBCS039 attenuated liver tissue damage in ALF mice.

### UBCS039 Inhibited the NF-κB Pathway and Inflammatory Reaction in Acute Liver Failure Mice

**FIGURE 3:**
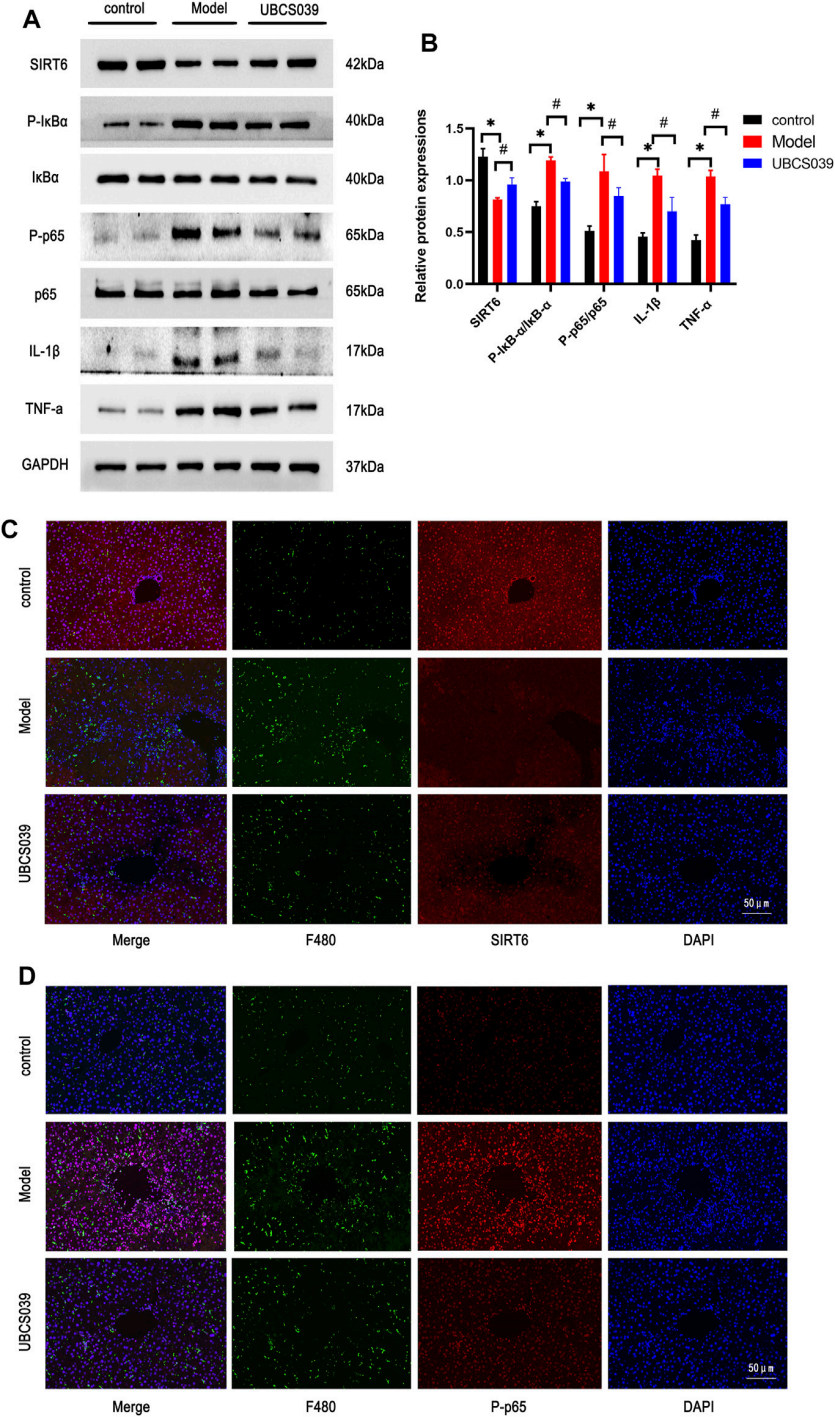
Effects of UBCS039 on the NF-κB pathway in liver tissues. **(A)** Western blotting was used to analyze the expressions of SIRT6, P-p65, p65, p-IKBa, IKBa, IL-1β, and TNF-α in the liver sample. **(B)** Densitometric analysis of relative proteins. **(C)** Immunofluorescent staining of liver sections with anti-F480 (green) and anti-SIRT6 (red). **(D)** Immunofluorescent staining of liver sections with anti-F480 (green) and anti-P-p65 (red). *p < 0.05, compared with the control group. #*p* < 0.05, compared with the model group. *n* = 5 per group. Data are represented as mean ± SD.

As a selective SIRT6 activator, UBCS039 reversed the decrease in SIRT6 in the liver of model mice. The NF-κB signaling pathway is determined as a critical inflammatory pathway involved in the pathogenesis of ALF, which triggers the transcription and release of inflammatory genes. In order to examine the impact of UBCS039 on the NF-κB pathway, we detected the relative proteins of the NF-κB pathway. Western blotting results revealed that the protein levels of p-P65 and p-IKBα were obviously elevated in the model group. The treatment with UBCS039 led to the repression of the NF-κB pathway. We next examined the anti-inflammation effect of UBCS039 on liver tissue of ALF. As expected, Western blotting confirmed that the UBCS039 group decreased the protein levels of IL-1β and TNF-α compared with model mice (Figures 3A,B). These results showed that UBCS039 participated the NF-κB pathway and inflammation in liver tissues of ALF mice. Finally, in order to detect the expression of SIRT6 and p-P65 in liver macrophages, the immunofluorescent staining of macrophage marker protein F4/80 and SIRT6 or p-P65 was performed. As expected, UBCS039 pretreatment decreased the F4/80 and p-P65 expressions compared with the model group (Figure 3D). The aforementioned data indicated that UBCS039 could inhibit liver inflammation and activation of macrophages.

### UBCS039 Reduced the Hepatocyte Apoptosis in Acute Liver Failure Mice

**FIGURE 4:**
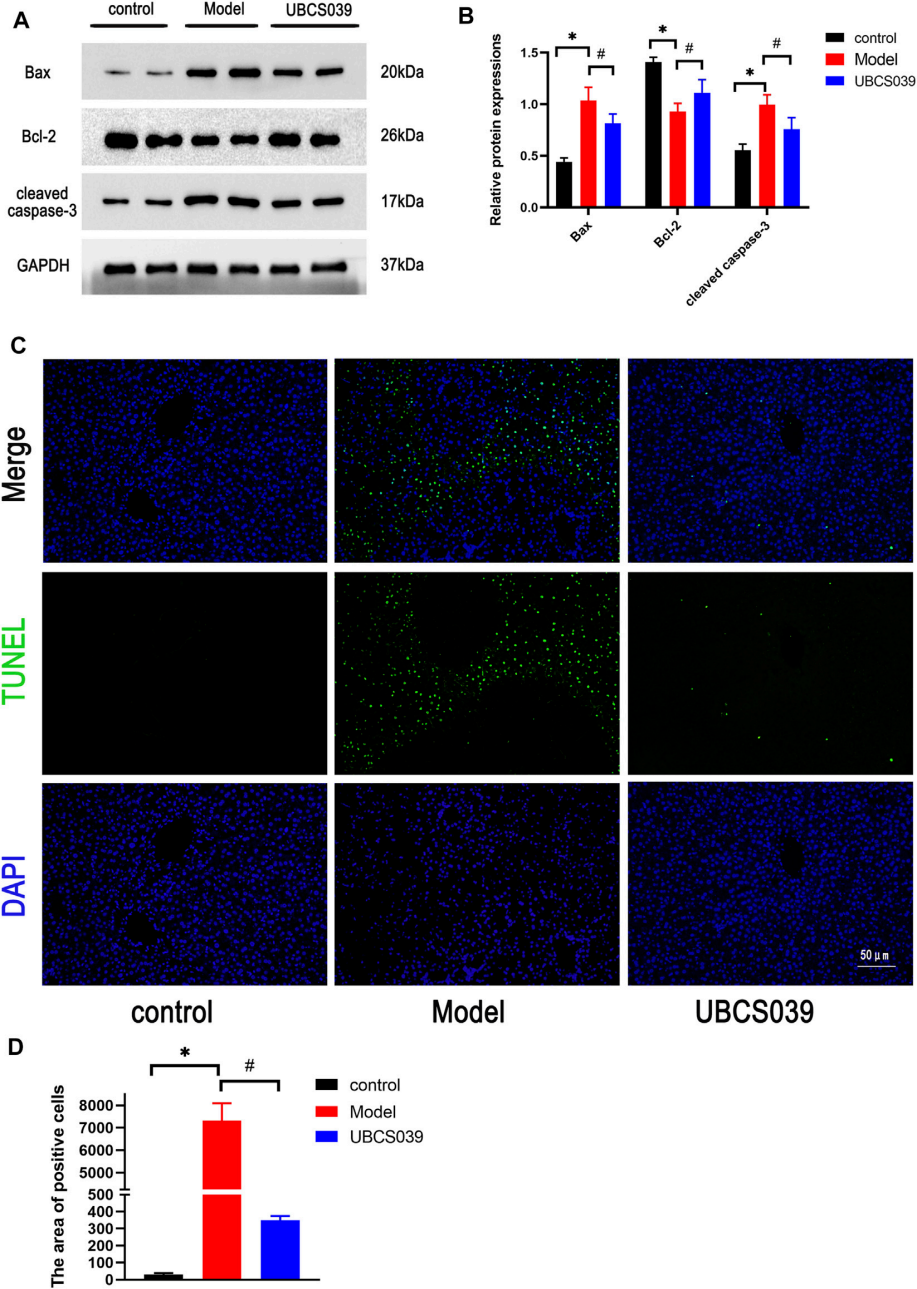
Effects of UBCS039 on hepatocyte death in liver samples. **(A)** Western blotting was used to analyze the expressions of SIRT6, Bax, Bcl-2, and cleaved caspase 3 in liver tissue. **(B)** Densitometric analysis of relative proteins. **(C)** TUNEL staining detected the number of apoptosis cells in liver sections. *p < 0.05, compared with the control group. #*p* < 0.05, compared with the model group. *n* = 5 per group. Data are represented as mean ± SD.

Hepatocyte apoptosis is a critical factor for the development of acute liver damage. To explore the protective effect of UBCS039 on hepatocyte apoptosis induced by TAA, relative proteins were measured by Western blot, and elevation of proapoptotic proteins (cleaved caspase 3 and Bax) and the downregulation of antiapoptotic Bcl-2 were detected in the model group. However, UBCS039 pretreatment partly reversed the upregulation of cleaved caspase 3 and Bax and the downregulation of Bcl-2 compared with the model group (Figures 4A,B). The results were further confirmed by TUNEL assay. As shown in Figure 4C, a larger number of apoptotic cells was observed in the model group, and UBCS039 pretreatment decreased the proportion of apoptotic cells (Figure 4C). These data suggested that UBCS039 reduced the apoptosis in the liver of ALF mice.

### UBCS039 Suppressed the Oxidative Stress in Acute Liver Failure Mice

**FIGURE 5:**
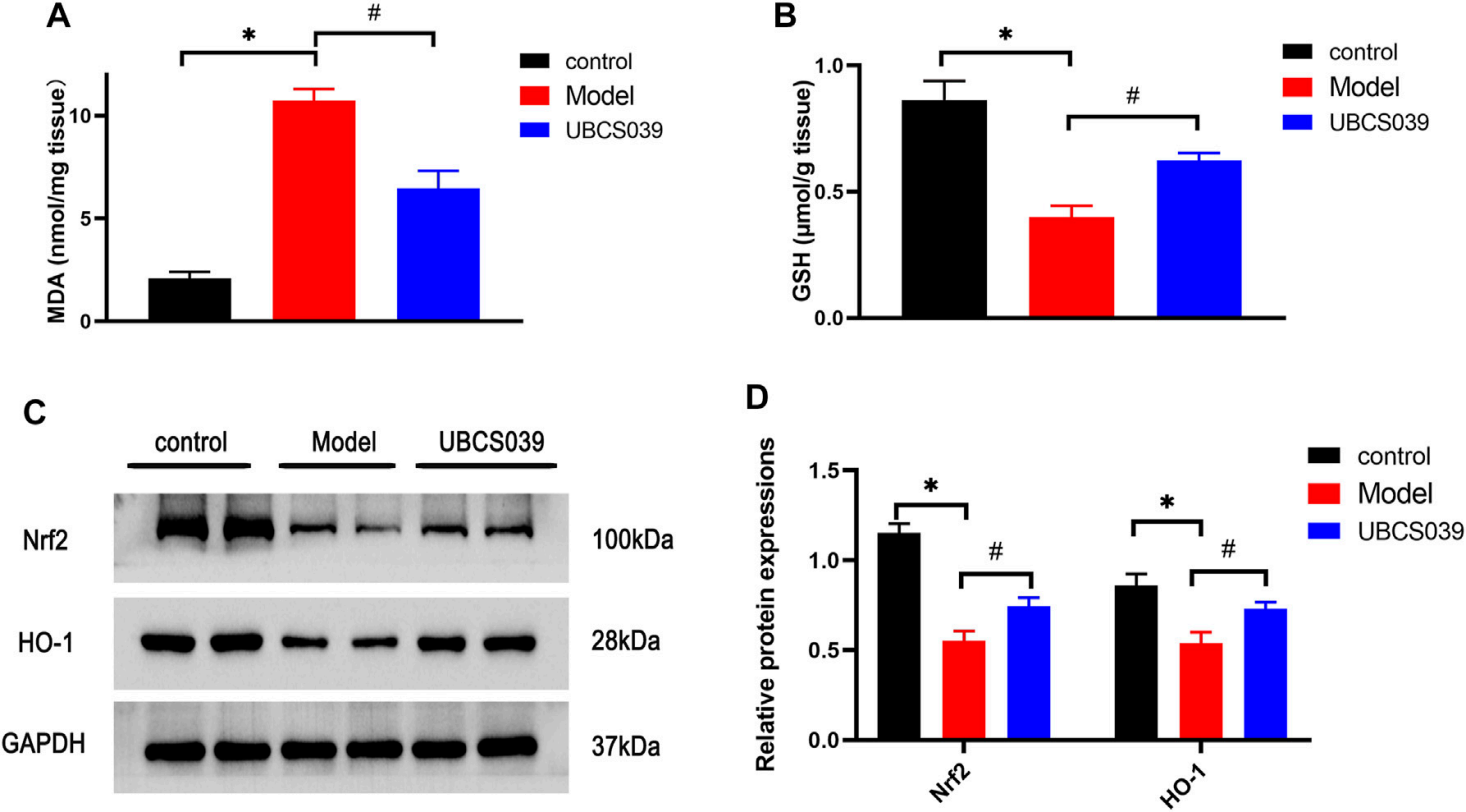
Effects of UBCS039 on oxidative stress in liver samples. **(A)** MDA level. **(B)** GSH level. **(C)** Western blot analysis of the Nrf2 and HO-1 levels in each group. **(D)** Densitometric analysis of the two proteins. *p < 0.05, compared with the control group. #*p* < 0.05, compared with the model group. *n* = 5 per group. Data are represented as mean ± SD.

Oxidative stress was considered as a critical mechanism involved in cell damage and death. We measured the related oxidative marker levels in the liver. TAA induced the high levels of MDA in liver tissue compared with the control group. Meanwhile, TAA reduced the levels of GSH in liver tissue compared with the control group. However, UBCS039 administration reduced the abnormal levels of oxidative stress markers after TAA exposure (Figures 5A,B). We next investigated the expressions of antioxidant proteins Nrf2 and HO-1 in the liver. The expressions of Nrf2 and HO-1 were significantly reduced in the model group compared with the control group. UBCS039 pretreatment upregulated the expression of the two proteins after TAA stimulation (Figures 5C,D). The results showed that UBCS039 could regulate oxidative stress damage in ALF mice.

### UBCS039 Inhibited Lipopolysaccharide-Induced Inflammatory Responses in RAW264.7 Macrophages by Regulating the NF-κB Pathway

**FIGURE 6:**
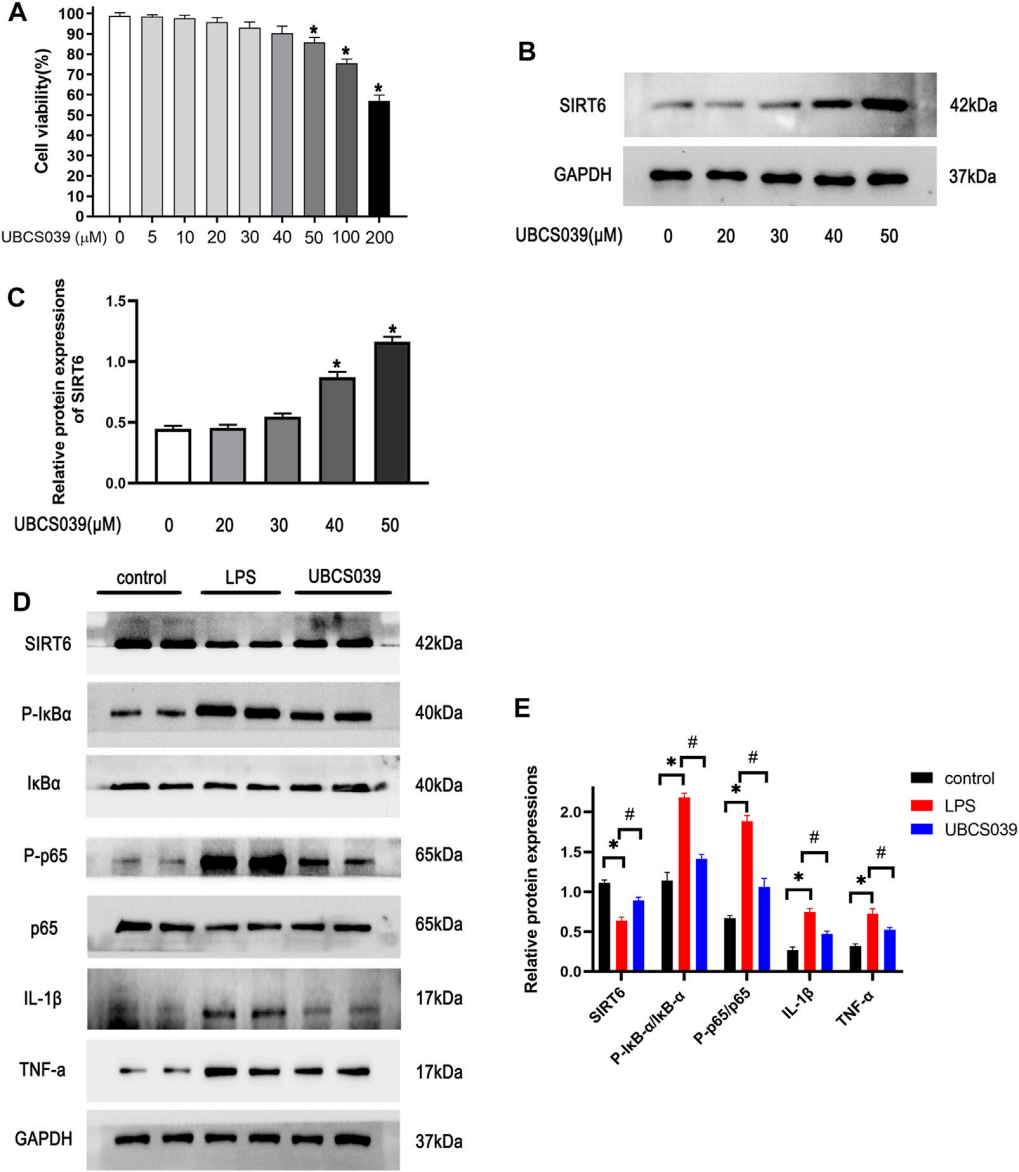
Effects of UBCS039 on the NF-κB pathway in LPS-induced RAW264.7 macrophages. **(A)** Toxic different doses of UBCS039 were by CCK-8 assay. **(B)** RAW 264.7 cells were exposed to UBCS039 (0, 20, 30, 40, and 50 μM) for 24 h; Western blot analysis of SIRT6 levels. **(C)** Densitometric analysis of SIRT6. **(D)** After pretreatment with 40 μM UBCS039 for 2 h, cells were added with 10 μg/ml LPS for 24 h. The relative proteins were detected by Western blot. **(E)** Densitometric analysis of these proteins. *p < 0.05, compared with the control group. #*p* < 0.05, compared with the model group. *n* = 3 per group. Data are represented as mean ± SD.

To identify non-toxic doses of the chemical, the suitable concentration of UBCS039 was first identified using the CCK-8 experiment. As shown in Figure 6A, high doses of UBCS039 (50 μM, 100 μM, and 200 μM) were cytotoxic to macrophages, with a cell viability lower than 90%. Thus, the maximum non-toxic dose of UBCS039 was 40 μM, and it was used in the cell experiment (Figure 6A). To verify the activation of SIRT6, the expression of SIRT6 was measured by Western blotting. We found that UBCS039 increased the SIRT6 protein levels in macrophages after 24-h LPS stimulation in a dose-dependent manner (Figures 6B,C). Subsequently, we examined the anti-inflammatory effect of UBCS039 on LPS-stimulated macrophages. The activation of the NF-κB pathway and the upregulation of inflammatory cytokines IL-1β and TNF-α were observed in the LPS group. UBCS039 treatment obviously downregulated the activation of the NF-κB pathway and inflammatory cytokines after LPS stimulation (Figures 6D,E). The *in vitro* data confirmed that UBCS039 could inhibit inflammatory responses in LPS-induced RAW264.7 macrophages.

### SIRT6 Knockdown Enhanced the Activation of the NF-κB Pathway in Lipopolysaccharide-Stimulated RAW264.7 Macrophages

**FIGURE 7:**
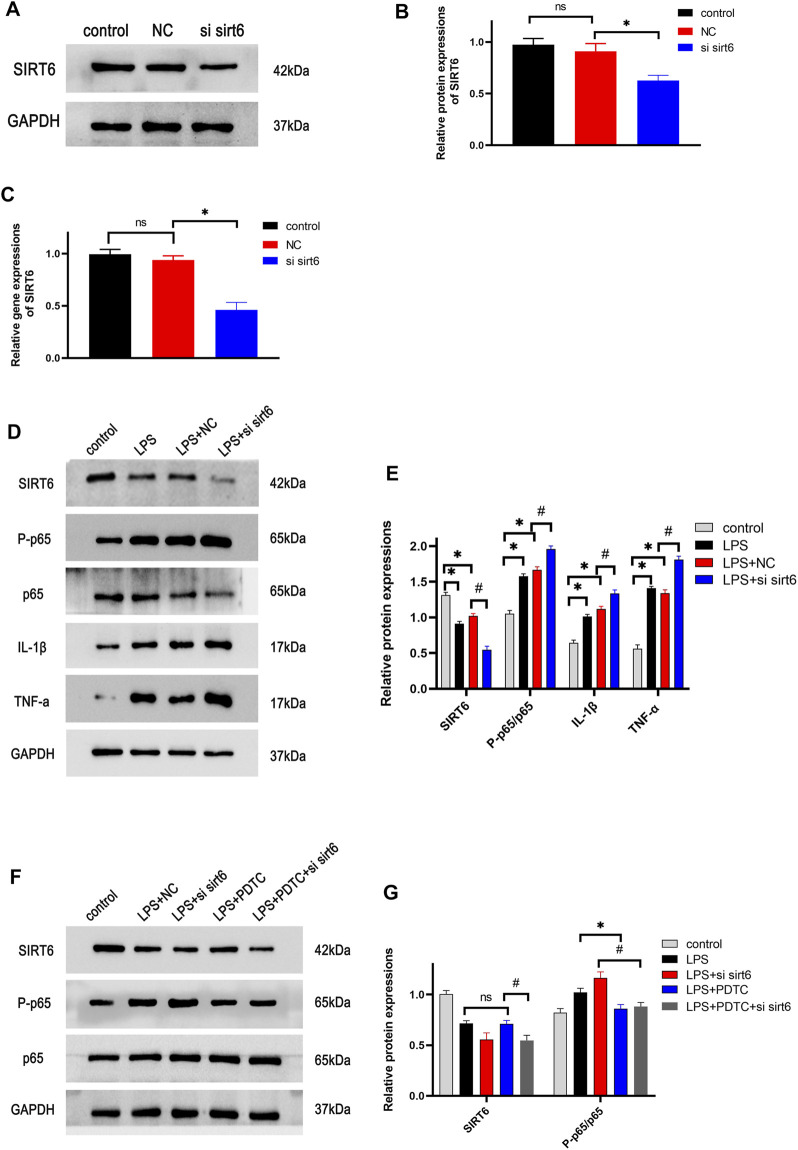
Effects of knockdown of SIRT6 on the NF-κB pathway in LPS-induced RAW264.7 cells. **(A)** After siRNA transfection for 24 h, the transfection efficiency of cells was detected by Western blotting **(B)** and RT-PCR **(C)**. **(D)** Cells were treated with siRNA and LPS, and the NF-κB pathway proteins were investigated by Western blotting. **(E)** Densitometric analysis of proteins. **(F)** NF-κB inhibitor, 50 μM PDTC was added in cells, the pathway protein expression was analyzed by treatment with siRNA and LPS. **(G)** Densitometric analysis of proteins. *p < 0.05, compared with the control group. #*p* < 0.05, compared with the model group. *n* = 3 per group. Data are represented as mean ± SD.

RAW264.7 macrophages were transfected with siRNA SIRT6 for 24 h, and the knockdown efficiency was verified by Western blotting and RT-PCR. These results indicated that siRNA SIRT6 effectively reduced the expressions of SIRT6 (Figures 7A–C). As expected, the downregulation of SIRT6 by siRNA increased the inflammatory response and the activation of the NF-κB pathway (Figures 7D,E). In order to further confirm the anti-inflammation of SIRT6, the NF-κB inhibitor PDTC was added to LPS-induced macrophages. Western blotting results showed that the LPS + PDTC group decreased the expression of p-P65 and inflammatory cytokines compared with the LPS group. Moreover, PDTC administration lowered the increase in p-P65 and inflammatory cytokines after LPS and siRNA SIRT6 stimulation (Figures 7F,G). These results suggested that SIRT6 knockdown promoted the inflammatory response in LPS-induced macrophages by the activation of the NF-κB pathway.

### SIRT6 Inhibited the Oxidative Stress in Thioacetamide-Induced Hepatocytes by Activating the Nrf2/HO-1 Pathway

**FIGURE 8:**
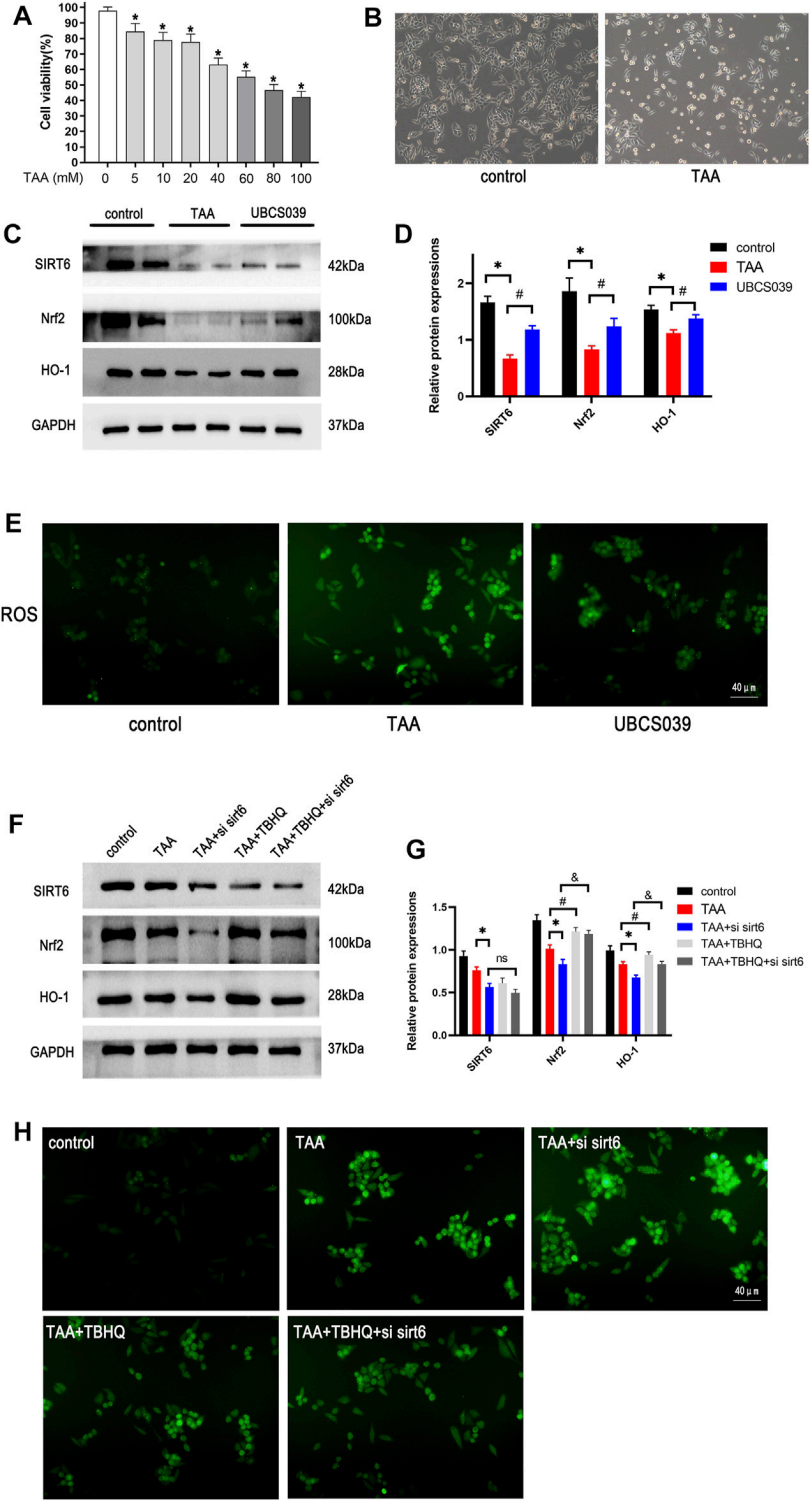
Effects of SIRT6 on the Nrf2/HO-1 pathway in TAA-induced hepatocytes. **(A)** Toxic different doses of TAA were by CCK-8 assay. **(B)** LO2 cells were exposed to 50 μM TAA; cellular morphology was observed under the light microscope. **(C)** Western blot analysis of SIRT6, Nrf2, and HO-1 levels in each group. **(D)** Densitometric analysis of these proteins. **(E)** ROS was observed under a fluorescence microscope. **(F)** Nrf2 activator, 20 μM TBHQ was added in cells, the Nrf2 pathway protein expression was analyzed by treatment with siRNA and TAA. **(G)** Densitometric analysis of these proteins. **(H)** ROS was observed under a fluorescence microscope. *n* = 3 per group. Data are represented as mean ± SD.

The proper cytotoxic dose of TAA on LO2 cells was measured by CCK-8 assay. As shown in Figure 8A, cell viability was 50% at the dose of 60 μM TAA. Abnormal cellular morphology was observed in TAA-induced hepatocytes (Figure 8B). We next measured the oxidative stress-related proteins in hepatocytes. Western blotting analysis revealed that TAA treatment reduced the expression of Nrf2 and HO-1 in LO2 cells at 24 h. The UBCS039 group showed increased expression of the two proteins compared with the TAA group (Figures 8C,D). In addition, the ROS levels were detected in this process. The results showed that the ROS levels were obviously upregulated in the TAA group compared with the control group. The UBCS039 group showed lower ROS levels than the TAA group (Figure 8E). Next, the Nrf2 activator TBHQ was used to further investigate the interaction of SIRT6 with the Nrf2/HO-1 pathway. As expected, TBHQ partly reversed the downregulation of Nrf2 and HO-1 in TAA-induced hepatocytes. Moreover, TBHQ administration upregulated the expression of Nrf2 and HO-1 after TAA and siRNA SIRT6 treatment (Figures 8F,G). Similarly, TBHQ administration reduced the ROS levels after TAA and siRNA SIRT6 treatment (Figure 8H).

## Discussion

**FIGURE 9:**
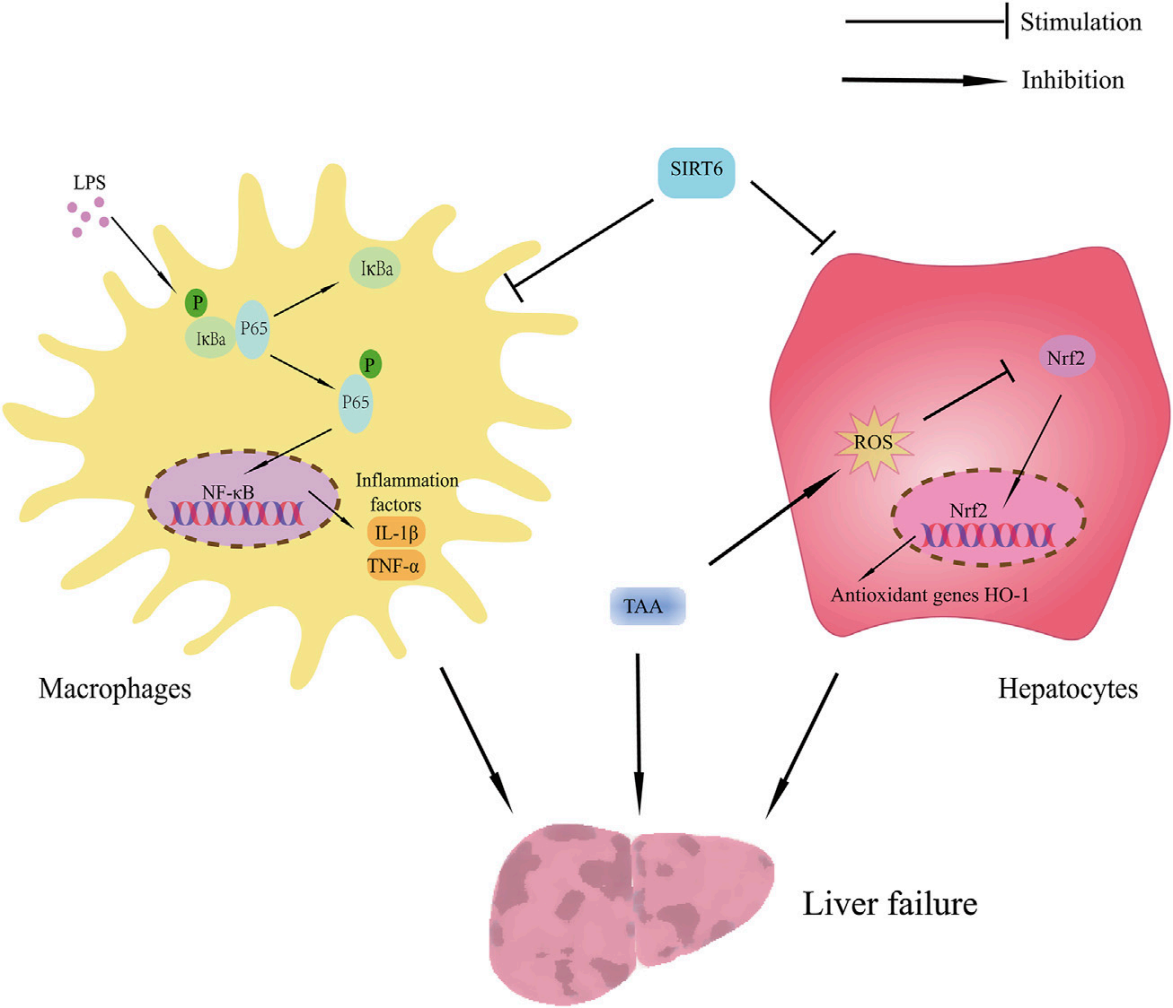
Schematic showing the benefit of SIRT6 on TAA-induced ALF mice. The protective effect of SIRT6 includes 1) upregulation of SIRT6 inhibiting inflammatory response by suppressing the NF-κB pathway and 2) upregulation of SIRT6 inhibiting oxidative stress by regulating the Nrf2/HO-1 pathway.

This research focused on the beneficial effect and possible mechanisms of SIRT6 on TAA-induced liver injury. The main finding includes the SIRT6 activator UBCS039 inhibited the inflammatory response by suppressing the NF-κB pathway in the TAA-induced ALF mice model and LPS-stimulated macrophages. Additionally, UBCS039 alleviated oxidative stress damage in hepatocytes *in vivo* and *in vitro* (Figure 9).

In the *in vivo* animal study, we explored the protective effect of UBCS039 on TAA-induced liver injury. Hepatic macrophages play a central role in the pathogenesis of acute liver failure (van der Heide et al., 2019), LPS-stimulated macrophages could secrete inflammatory cytokines and were widely used to establish inflammatory cell models (Rossol et al., 2011; Utaipan et al., 2018). Thus, in *in vitro* cell experiments, LPS-induced macrophages were used to explore the relative mechanisms of how SIRT6 regulates the inflammation. In addition to inflammation, we detected the direct effect of hepatotoxic drug TAA on hepatocytes *in vitro*.

Although sirtuins belong to class III deacetylases, different members of sirtuins have different subcellular localizations, catalysis activities, and effective targets. As a chromatin-bound protein, SIRT6 is located in the nuclear and has strong enzymatic activities, such as deacetylase activity and mono-ADP-ribosyl transferase activity (Jiao and Gong, 2020). High expression of SIRT6 was confirmed in diverse organs and tissues, including the muscles, brain, liver, and heart (Michishita et al., 2005). SIRT6 is not only widely distributed but also functionally diverse, such as inflammation, metabolism, aging, and oxidative stress (Beauharnois et al., 2013). Accumulated evidence has been reported that SIRT6 involved in regulating inflammation. One study has reported that SIRT6 deficiency increased the levels of pro-inflammatory mediators (TNF-α, IL-6, and IL-1β) in podocytes (Liu et al., 2017). Recently, a study showed that the upregulation of SIRT6 lowered the levels of inflammatory factors TNF-a, IL-1β, and IL-6 in LPS-injured PC12 cells (Zhaohuia and Shuiua 2020). SIRT6 transgenic mice showed lower levels of pro-inflammatory cytokines like TNF-a, IL-1β, and IL-6 in the dextran sulfate sodium salt (DSS)-induced colitis model than normal mice (Xu et al., 2020). In addition, previous studies have reported that the SIRT6 activator UBCS039 could effectively upregulate SIRT6 and inhibit the activation of macrophages (Zou et al., 2021; Wang et al., 2022). Although SIRT6 plays an important role in several disease models, there is no report on the effect of SIRT6 on the liver failure model. Our current study confirmed that the upregulation of SIRT6 by UBCS039 led to the decrease in pro-inflammatory cytokines in TAA-induced ALF and LPS-induced macrophages, which also indicated the anti-inflammatory effect of SIRT6.

The transcription factor NF-κB is considered a critical signal molecule, which mediates the pro-inflammatory pathway by regulating the genetic transcription and the release of pro-inflammatory cytokines (Lawrence 2009). In the physiological state, NF-κB is a dimeric protein, which consists of P65 and P50 subunits, and it is inactive in the cytoplasm when bound to IκBα. When P65 is phosphorylated, it moves into the nucleus and regulates gene transcription (Viatour et al., 2005). The activation of the NF-κB pathway was found in various inflammatory diseases, including liver failure. Several animal experiments revealed that inhibiting the activation of the NF-κB pathway attenuated the liver inflammation (Sun and Karin, 2008; Muriel 2009). In accordance with earlier studies, this study also confirmed that the NF-κB pathway was activated in liver tissue of TAA-induced ALF mice. Increasing evidence supported the anti-inflammatory properties of SIRT6. However, the potential mechanism remains unknown. Some studies revealed that the anti-inflammatory mechanism of SIRT6 was *via* modulating target gene transcription, especially NF-κβ. Nonetheless, it was also discovered that SIRT6 did not directly deacetylate P65 but inhibited NF-κβ activity by histone H3K9 deacetylation (Kawahara et al., 2009; Mendes et al., 2017). A recent animal experiment study showed that SIRT6 overexpression suppressed the phosphorylation of NF-κB in DSS-induced colitis (Xu et al., 2020). Another experiment showed that SIRT6 attenuated Ang II-induced NF-κB activation in vascular aging by suppressing the NF-κB pathway (Liu X. et al., 2021).

However, there is no relative study on whether SIRT6 regulates the liver inflammation by inhibiting the NF-κB pathway in liver failure. In order to confirm the hypothesis, the SIRT6 activator UBCS039 was used in TAA-induced mice. As expected, our findings suggested that the upregulation of SIRT6 hindered the activation of NF-κB signaling in ALF.

Apart from inflammation, oxidative stress-induced hepatocyte damage is an important pathogenetic factor of liver failure (Thiel et al., 2014). The abnormal levels of oxidative stress parameters were found in several liver failure models (Wei et al., 2014; Chen et al., 2016; González-Ponce et al., 2020). Similarly, the abnormal oxidative marker levels were also detected in TAA-induced ALF mice. It is well-known that Nrf2 is a critical nuclear transcriptional factor. Once activated, Nrf2 can regulate the antioxidant genes transcription, including ARE and HO-1 (Zhang et al., 2019). A large number of studies demonstrated that SIRT6 could activate the expressions of Nrf2 and mediate antioxidant effects. An *in vitro* experiment has reported that the overexpression of SIRT6 suppressed the oxidative stress in angiotensin II-induced vascular endothelial cells by increasing the nuclear expression of Nrf2. In the meantime, Nrf2 siRNA attenuated the protective effect of SIRT6 (Yang et al., 2019). Another study showed that the overexpression of SIRT6 increased nuclear NRF2 expression and reduced oxidative stress in the cerebral ischemia/reperfusion (I/R) model. But, this beneficial effect of SIRT6 was reversed in Nrf2 knockout mice (Zhang et al., 2017). However, the regulatory effects of SIRT6 in oxidative stress of liver failure are still unknown. Our results showed that the upregulation of SIRT6 ameliorated the levels of Nrf2 and HO-1 in TAA-induced hepatic injury *in vitro* and *in vivo*. Moreover, the Nrf2 activator TBHQ decreased the inhibition of the Nrf2 pathway in liver injury.

In conclusion, our study indicated that SIRT6 activator UBCS039 protected the liver against TAA-induced damage in ALF mice. The potential mechanisms include inhibiting the oxidative stress and liver inflammation. The upregulation of SIRT6 might be a potential therapeutic target for ALF, and more related studies are needed to confirm the therapeutic value in ALF.

## Data Availability

The original contributions presented in the study are included in the article/Supplementary Material, further inquiries can be directed to the corresponding author.
