# Supplementary material for: SIRT6 Activator UBCS039 Inhibits Thioacetamide-Induced Hepatic Injury In Vitro and In Vivo
Source: Front Pharmacol. 2022 Apr 20;13:837544. doi: 10.3389/fphar.2022.837544 (PMC9065480; doi:10.3389/fphar.2022.837544)
Supplement: Supplementary file 1 [file Image1.pdf]

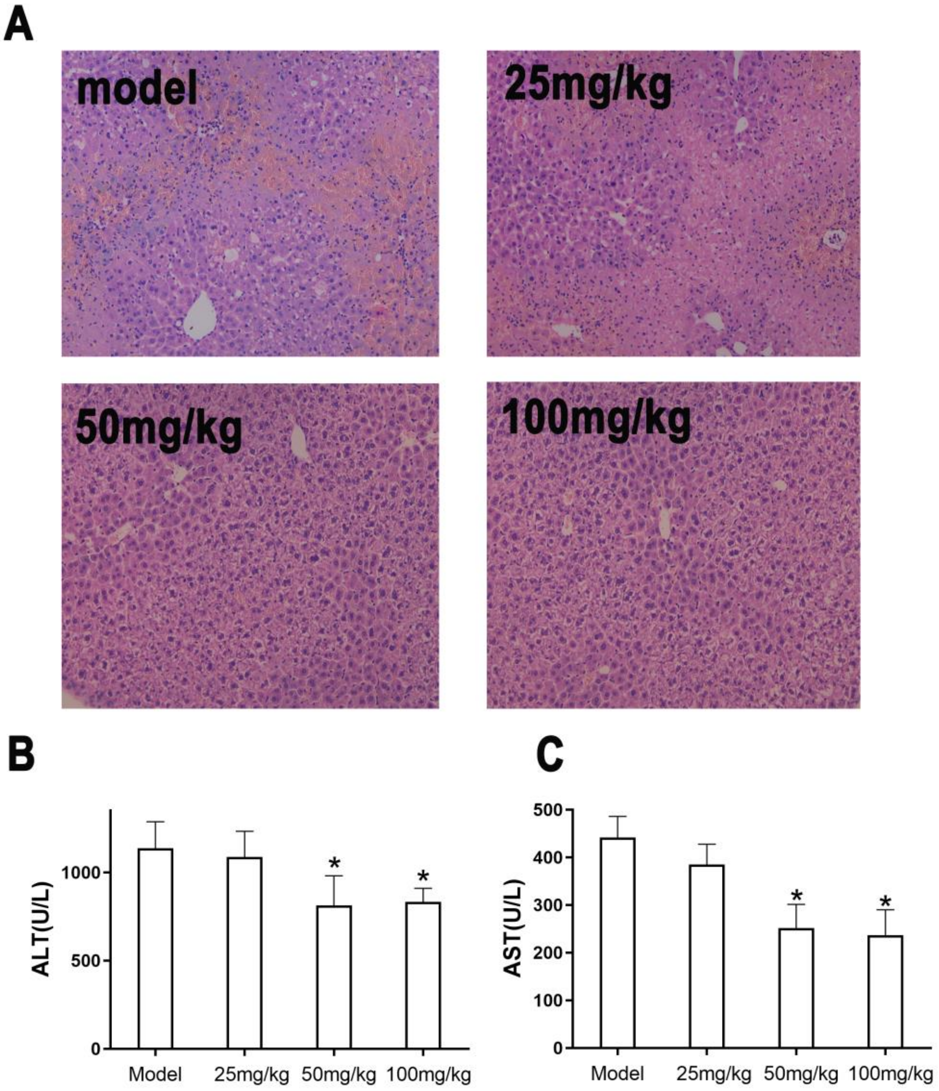

Figure S1. Effect of different dosages UBCS039 (25mg/kg, 50mg/kg, 100mg/kg) on liver structure and function in ALF mice. (a)The histological changes of liver were observed by HE staining. (b) The ALT and AST levels. n=3 per group. The data represent the means ± SD. $P < 0.05$, compared with the control group.
